# Long-term evaluation of the seroprevalence of SARS-CoV-2 IgG and IgM antibodies in recovered patients: a meta-analysis

**DOI:** 10.1186/s12879-023-08425-3

**Published:** 2023-07-01

**Authors:** Qiu Li, Lu Chen, Fen Li, An He

**Affiliations:** 1 Laboratory Medicine Center, Chenzhou First People’s Hospital, Chenzhou, 423000 P.R. China; 2 Baoshan Community Hospital, Chenzhou, 424400 P.R. China

**Keywords:** SARS-CoV-2, COVID-19, IgG, IgM, Seroprevalence, Recovered patients

## Abstract

Estimating severe acute respiratory syndrome coronavirus 2 (SARS-CoV-2) -specific immunoglobulin G (IgG) immunoglobulin M (IgM) antibodies are increasingly important for tracking the spread of infection and defining herd immunity barrier and individual immunization levels in the ongoing coronavirus disease 2019 (COVID-19) pandemic. Therefore, we conducted the present systematic review and meta-analysis to evaluate the seroprevalence of SARS-CoV-2 IgM and IgG antibodies of recovered COVID-19 patients in long-term follow-up studies. A systematic search of the MEDLINE, Embase, COVID-19 Primer, PubMed, CNKI, and the Public Health England library databases was conducted. Twenty-fourth eligible studies were included. Meta-analysis showed that 27% (95%CI: 0.04–0.49) and 66% (95%CI:0.47–0.85) were seropositive for SARS-CoV-2 IgM and IgG, respectively, while in long-term 12 months following up studies, the seroprevalences of IgM antibody (17%) decreased and IgG antibody (75%) was higher than 6 months follow-up patients. However, due to the limited number of relevant studies, the high level of heterogeneity, and the large gap in studies conducted, the findings of our study may not accurately reflect the true seroprevalence status of SARS-CoV-2 infection. Nevertheless, sequential vaccination or booster immunization is considered to be a necessary long-term strategy to sustain the fight against the pandemic.

## Introduction

The novel coronavirus disease (COVID-19) is a highly contagious disease caused by the SARS-CoV-2 virus, leading to significant morbidity and mortality in a proportion of patients. According to the World Health Organization (WHO), as of 30 May 2023, the cumulative number of confirmed COVID-19 cases caused by the novel SARS-CoV-2 worldwide reached over 676.66 million in more than 180 countries, and the cumulative number of deaths reached over 6.88 million. (https://www.arcgis.com/apps/opsdashboard/index.html#/bda7594740fd40299423467b48e9ecf6). In patients who survive COVID-19, a certain degree of immunity against SARS-CoV-2 is expected. The exact proportion of the population that needs to develop immunity against SARS-CoV-2 to ensure herd immunity is unknown, most experts have suggested that at least 60–80% of the population would need to be immune via either natural infection or immunization [1, 2].

Immunoglobulin G (IgG) immunoglobulin M (IgM) antibodies play crucial roles in the long-term follow-up of COVID-19 patients, providing invaluable insights into the dynamics of immunity and disease progression. Therefore, knowing the seroprevalence of anti-SARS-CoV-2 antibodies in COVID-19-recovered patients is important, and systematic screening for antibodies against SARS-CoV-2 is an important tool in the surveillance of the pandemic [3]. Following COVID-19 infection, the human immune system produces a range of immune responses, including the production of IgG and IgM antibodies. IgM antibodies emerge early during immune responses, while IgG antibodies typically appear later and exist in human bodies for months. The levels of IgG and IgM antibodies against nucleoprotein and surface spike protein receptor binding domain increased gradually after symptom onset, and both showed correlation with virus neutralization titer. A substantial decline in IgG and IgM antibodies was reported over 3 months post-infection, yet other studies showed a stable antibody level after 6 to 12 months post-infection [4–6]. The seroconversion rate of IgG (90%) antibodies was higher than that of IgM (32%) antibodies after the onset of COVID-19, in contrast to the persistence of IgG antibodies, but also reveal IgG loss in around 50% of COVID-19 survivors 10 months after their recovery [7]. However, the duration and effect of IgG and IgM antibodies and their ability to resist reinfection are unclear, and the overall seroprevalence of antibodies in long-term follow-up is poorly understood. Our objectives were to investigate the seroprevalence of IgG and IgM SARS-CoV-2 antibodies of recovered COVID-19 patients in long-term follow-up studies (follow-up time ≥ 6 months). Hopefully, these results will contribute to the full acceptance of COVID-19 vaccines in order to establish a herd immunity barrier and strengthen the level of immunization, especially in medical resource-limited settings.

## Methods

The review was conducted following Systematic Reviews and Meta-Analyses (PRISMA) guidelines [8].

### Search strategy and selection criteria

Keyword-structured searches were performed in MEDLINE, Embase, COVID-19 Primer, PubMed, Chinese Knowledge Infrastructure (CNKI), and the Public Health England library. Articles published between 01/07/2020 and 25/05/2022 were researched. The Boolean search strategy was as follows: ((COVID-19 OR SARS-CoV-2) AND (IgG OR IgG Antibody OR immunoglobulin M OR IgM OR IgM Antibody OR immunoglobulin M OR convalescent plasma OR convalescent serum OR antibody). The search terms were broad to encompass all applicable studies. The outcome of interest in this study is the seroprevalence of IgG and/or IgM in COVID-19 recovered patients with at least 6 months of follow-up. Accordingly, original studies reported information on the serum IgG and/or IgM levels were considered eligible for inclusion, whilst comments, case reports, editorials, and reviews were excluded. We excluded studies without original data, if data could not be extracted or calculated from the original article, or if the titer cut-offs used were not comparable to other studies.

### Inclusion and exclusion criteria

The inclusion criteria were: (1) COVID-19 patients confirmed by RT-PCR. (2) Reported the seroprevalences of IgG and/or IgM antibodies. (3) At least 6 months’ follow-up period. The exclusion criteria were: (1) Animal trials, case reports, and editorial materials. (2) Commentaries or opinion pieces not presenting any primary data. (3) Incomplete full text or non-conforming data. (4) Different studies with reduplicated populations.

### Data extraction

Two researchers independently screened the literature, and extracted and cross-checked the data. Using a standardized data collection form, information was extracted from the selected trials. Data included author, year, country, study design, age, gender, number of participants, the severity of symptoms (asymptomatic, mild, moderate, severe), serum IgG and/or IgM levels, and weeks/months elapsed since infection.

### Quality assessment

One researcher collected data from each report and the other reviewer independently checked the work. Any disagreement was resolved through discussion or judged by a third researcher [9, 10]. The MINORS (Methodological Index for Non-Randomized Studies) was used to assess the quality of the existing literature.

All the MINORS scores of included literatures were greater than 20, indicating that all the studies included in this meta-analysis were of relatively high quality and low risk of bias.

### Statistical analysis

When data were reported as medians and inter-quartile range, we converted them into approximate sample mean and standard deviation according to the method improved by Luo et al [11] and Wan et al [12] to pool results in a consistent format.

All the analyses were performed using Stata statistical package version 16.0 software. The results for continuous variables were presented in standard mean differences (SMD), seroprevalences of IgG and IgM were extracted to measure the pooled estimates, a single-arm meta-analysis was conducted to obtain the pooled prevalence and 95% confidence interval (95% CI) [10]. The Chi-square test or Cochrane Q test was used to calculate heterogeneity, and I^2^ < 50% and *P* > 0.10 were defined as non-significant heterogeneity, and such data were evaluated using the fixed effect model; otherwise, the random effect model was chosen, which based on our understanding of whether or not all included trials share a common effect size and not only on results of tests for statistical heterogeneity.

In addition, the publication bias of literature was evaluated by Funnel plot and Egger’s test, and p < 0.05 was considered statistically significant, indicating that publication bias was not excluded.

## Results

### Search strategy

**Fig. 1:**
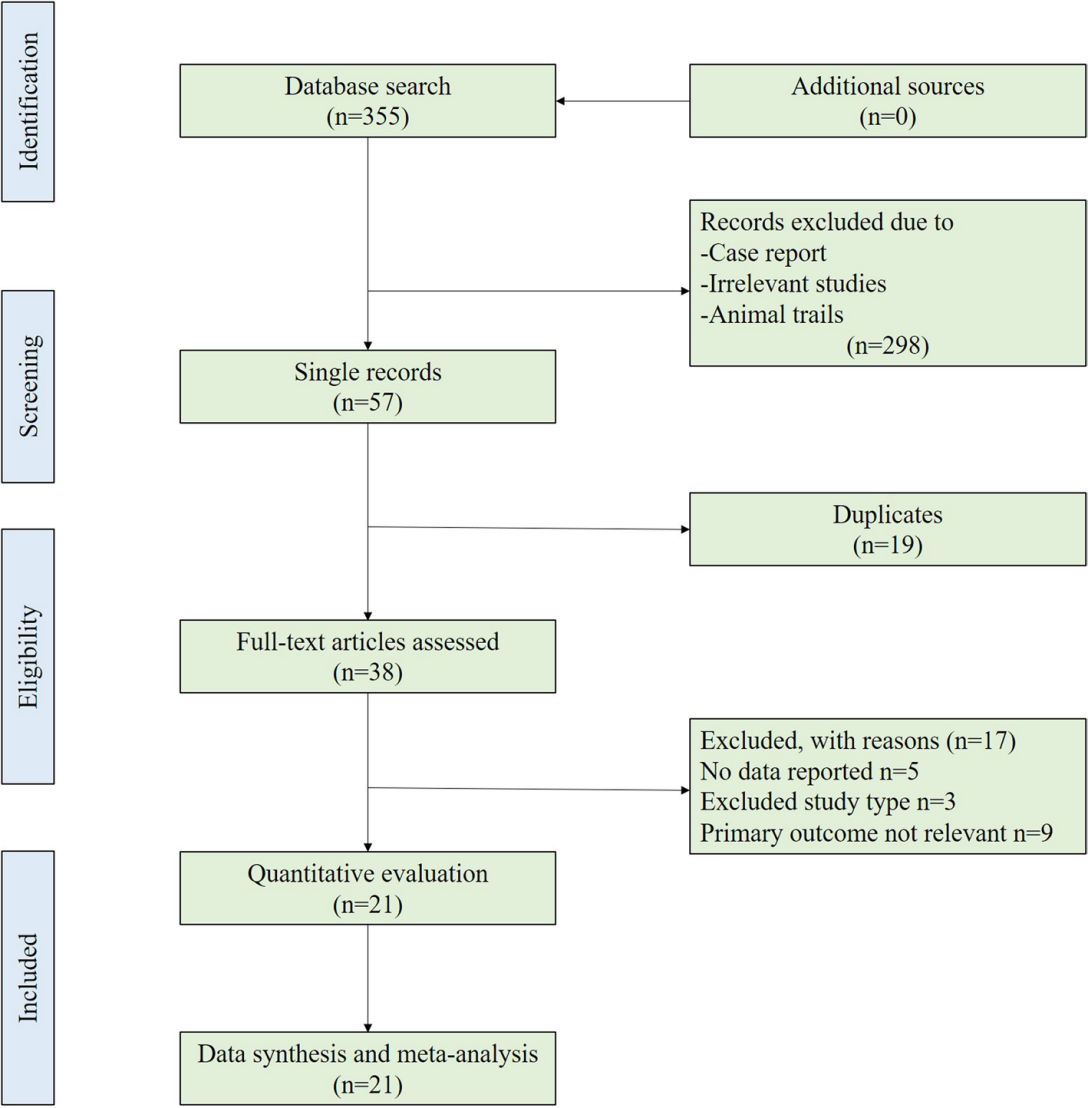
Flow diagram of the studies selection process

The search identified 355 reports. After screening titles and abstracts 78 full-text articles were assessed for eligibility, resulting in 21 studies that met the criteria for inclusion in the analysis. The flow chart of the search strategies is summarized in Fig. 1.

### Characteristics of the included studies

The baseline characteristics of the 21 studies such as first author’s name, year of publication, country, sample sizes, gender, age, patients’ condition, basic illness, trial duration, and the number of SARS-CoV-2 IgG and IgM cases are shown in Table 1.

**Table 1:** Study characteristics of the 21 studies included

First author	Year	duration of study	Country	Sample size	Gender (male/ female)	Age (Mean ± SD)	Disease severity (asymptomatic infection/light/severe/critical)	Underlying disease (yes/no)	Follow-up duration	Case of IgG positive (n)	Case of IgM positive(n)	Vaccines (type/doses)
Wang [13]	2021	3 months	China	43	22/21	44.91 ± 15.67	0/23/11/9/0	1/42	6 months	42	7	NA
Zhao [14]	2021	3 months	China	41	18/23	47.83 ± 12.95	0/32/0/9/0	NA	12 months	33	NA	NA
Liang [15]	2021	1 month	China	181	NA	NA	0/126/47/0/0	NA	6 months	167	51	inactivated vaccines/1
Shen [16]	2021	1 month	China	110	50/60	50.3 ± 12.9	0/14/80/16/0	48/62	6 months	39	14	inactivated vaccines/1
Zhang [17]	2022	5 months	China	9	NA	NA	NA	NA	6 months	9	4	NA
Xu [18]	2021	1 month	China	11	4/7	50.09 ± 9.77	0/11/0/0/0	NA	12 months	11	0	NA
Jia [19]	2021	1 month	China	170	75/95	NA	0/75/80/14/1	50/120	10 months	79	8	inactivated vaccines/1
Ren [20]	2021	3 months	China	68	32/36	43.56 ± 2.1	0/75/80/14/2	NA	6 months	33	0	NA
Zhan [21]	2021	1 month	China	105	54/51	NA	0/12/85/6/2	NA	6 months	2	0	NA
Yan [22]	2021	2 months	China	121	50/71	NA	0/0/0/121	37/84	12 months	55	2	inactivated vaccines/1
Anat [23]	2021	NA	Israel	392	284/108	NA	NA	NA	6 months	59	NA	NA
Hamza [24]	2021	NA	France	83	59/24	NA	0/0/0/83	NA	6 months	74	NA	NA
Lin [25]	2021	NA	China	59	31/28	41	5/16/38/0	NA	9 months	58	NA	NA
Mar [26]	2021	NA	Spain	80	49/31	NA	NA	49/31	12 months	73	NA	NA
Feng [27]	2021	1 month	China	204	NA	NA	1/12/162/29	NA	12 months	100	103	inactivated vaccines/1
Zhao [28]	2021	NA	China	67	NA	NA	0/2/30/35	NA	12 months	59	12	NA
Maddalena [7]	2021	NA	Italy	546	257/289	53.1	NA	NA	10 months	257	492	NA
Zhu [29]	2021	NA	China	64	NA	46.72	NA	NA	7 months	50	19	NA
Guo [30]	2022	NA	China	1096	587/509	58	0/0/289/807	NA	12 months	1032	26	NA
Julia [31]	2021	NA	Germany	412	177/235	NA	36/209/148/15	166/239	10 months	316	NA	NA
Larry [32]	2021	NA	USA	370	NA	41	NA	NA	6 months	294	92	NA

*NA* Not available

### The seroprevalences of IgG antibodies in recovered COVID-19 patients

**Fig. 2:**
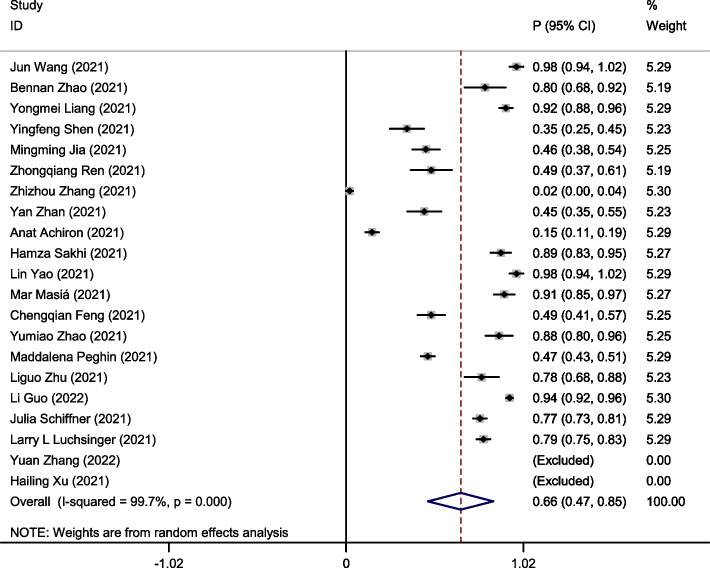
Forest plot showing the seroprevalences of IgG antibodies in recovered COVID-19 patients. Effect estimates are reported with 95% confidence intervals (CIs) and *p*-values

**Fig. 3:**
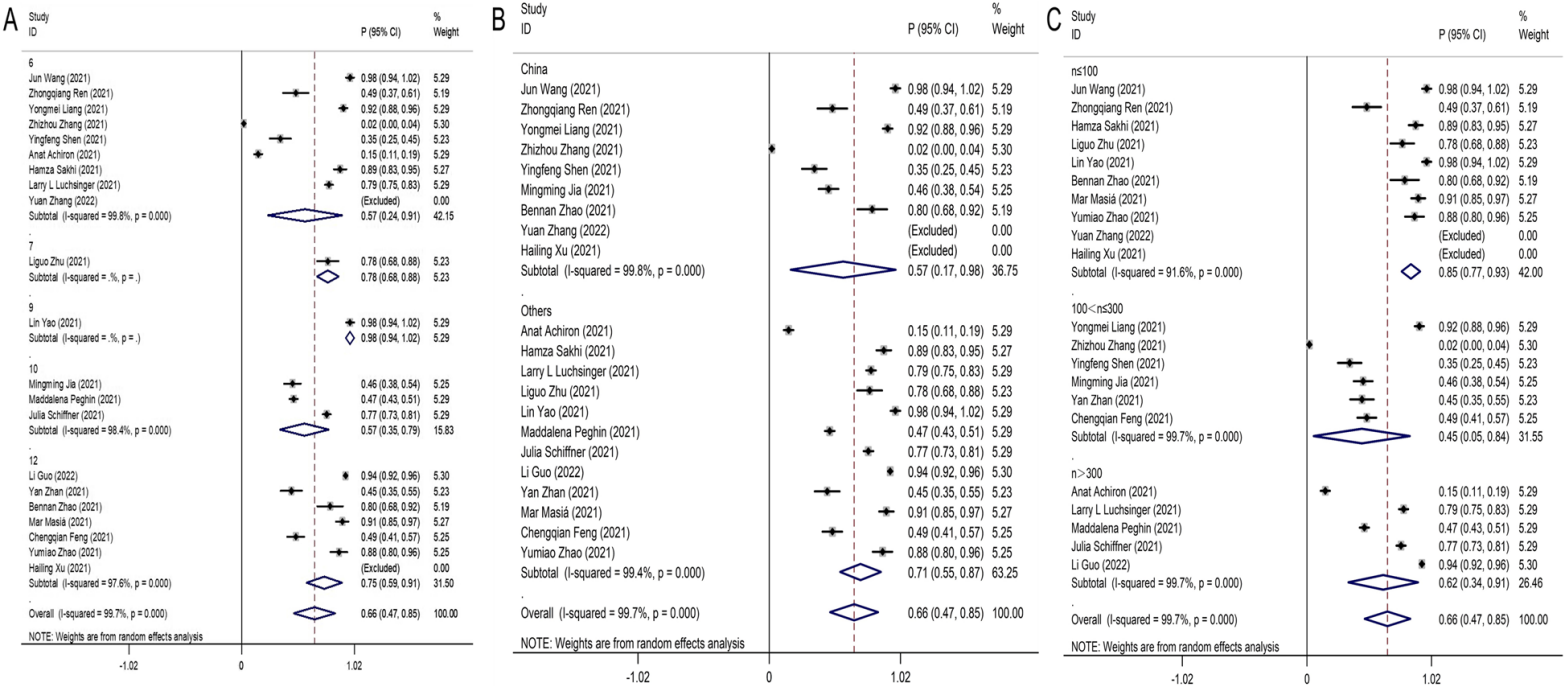
Subgroup analysis for follow-up time (**A**), country (**B**), sample size (**C**) estimating the IgG antibodies seroprevalences of recovered COVID-19 patients in the long-term follow-up studies (follow-up time ≥ 6 months). Effect estimates are reported with 95% confidence intervals (CIs) and *p*-values

A random effects model was used to pool data due to the large heterogeneity among included studies (I^2^ = 99.7%, P < 0.001). Pooling of the data showed the seroprevalences of IgG antibodies was 66% (95%CI:0.47–0.85) in recovered COVID-19 patients with long-term follow-up (follow-up time ≥ 6 months) (Fig. 2). To clarify the heterogeneity, subgroup analysis for follow-up months (Fig. 3A), country (Fig. 3B), sample size (Fig. 3C) was performed. The number of individual studies in 7- and 9-months following up was insufficient that could be pooled in subgroup analyses. Similar trends were observed in the subgroup analysis of the different follow-up duration. Interestingly, the seroprevalences of IgG antibodies in 12 months follow-up of recovered COVID-19 patients was 75% (95%CI:0.59–0.91), which was higher than 6 months follow-up (57%; 95%CI:0.24–0.91) and 10 months follow-up group (0.57%; 95%CI:0.34–0.80).

**Fig. 4:**
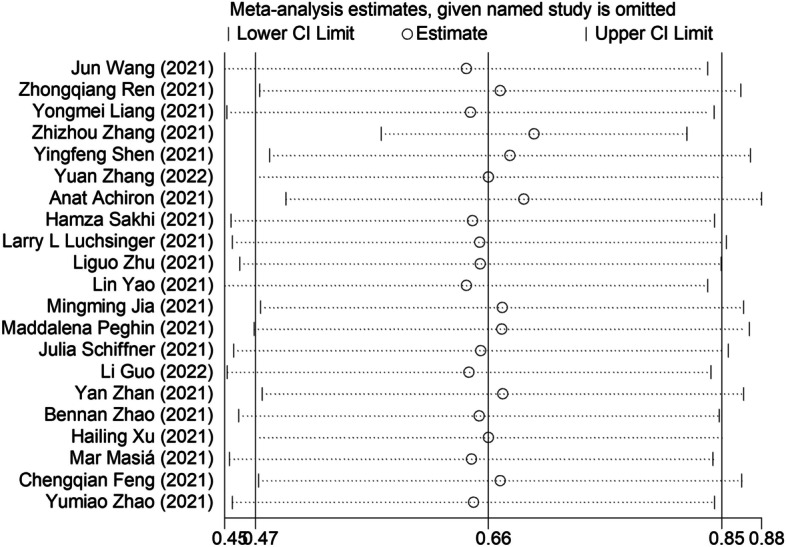
Sensitivity analysis of IgG antibodies seroprevalence. Sensitivity analysis was performed by sequential omission of individual studies

**Fig. 5:**
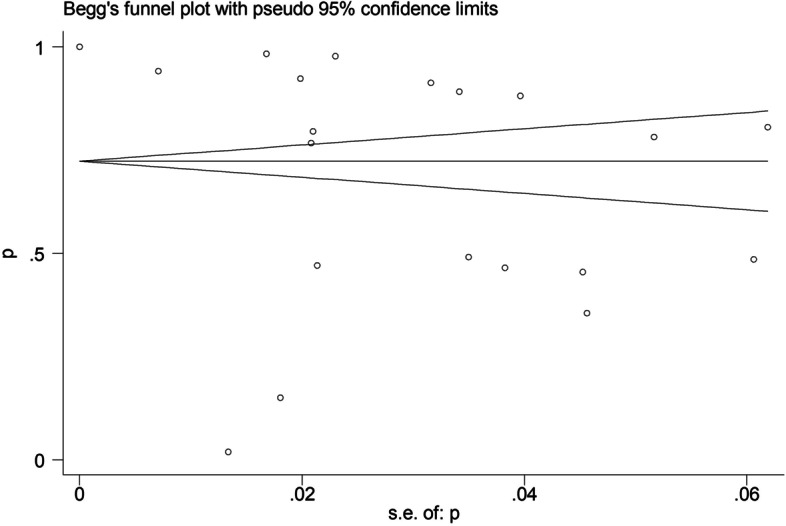
Publication bias of IgG antibodies seroprevalence

The sensitivity analysis was performed by omitting one study at a time to assess the robustness of the overall results. The *p* values for Egger’s (*p* = 0.399) tests indicated that publication bias was not present (Fig. 4), and the visual inspection of the funnel plot also did not reveal any asymmetry (Fig. 5).

### The seroprevalences of IgM antibodies in recovered COVID-19 patients

**Fig. 6:**
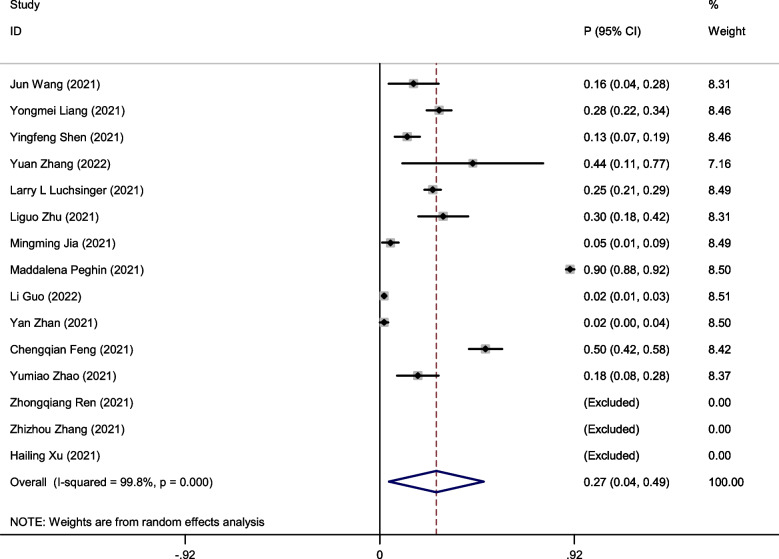
Forest plot showing the seroprevalences of IgM antibodies in recovered COVID-19 patients. Effect estimates are reported with 95% confidence intervals (CIs) and *p*-values

**Fig. 7:**
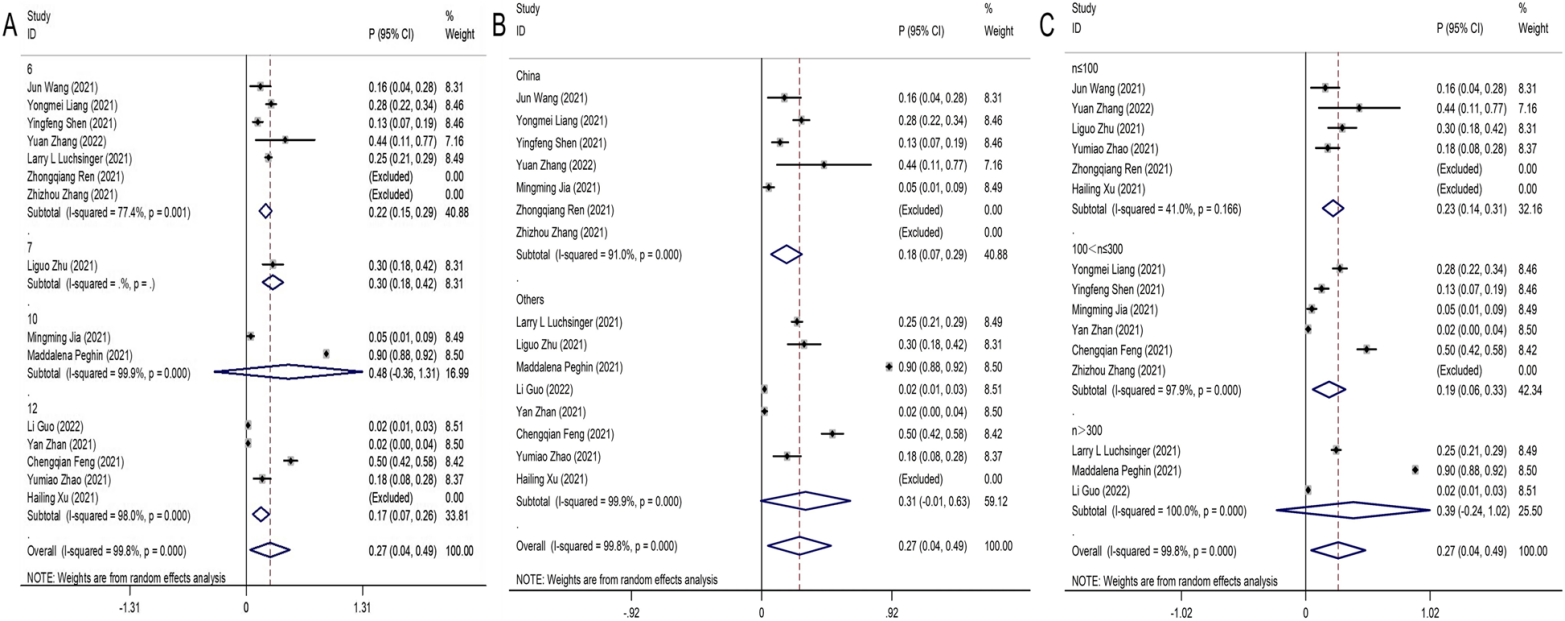
Subgroup analysis for follow-up time (**A**), country (**B**), sample size (**C**) estimating the IgM antibodies seroprevalences of recovered COVID-19 patients in the long-term follow-up studies (follow-up time ≥ 6 months). Effect estimates are reported with 95% confidence intervals (CIs) and *p*-values

A random effects model was used to pool data due to the large heterogeneity among included studies (I^2^ = 99.7%, *P* < 0.001). Pooling of the data showed the seroprevalences of IgM antibodies was 27% (95%CI: 0.04–0.49) in recovered COVID-19 patients with long-term follow-up (follow-up time ≥ 6 months) (Fig. 6). To clarify the heterogeneity, subgroup analysis for follow-up months (Fig. 7A), country (Fig. 7B), sample size (Fig. 7C) was performed. Unfortunately, the number of studies in 7 months follow-up was insufficient to be pooled in subgroup analyses. The heterogeneity of 6 months follow-up was significantly decreased (77.4%, *P* = 0.001) and the seroprevalences of IgM antibodies was 22% (95%CI: 0.15–0.29). No statistically significant were found in the seroprevalences of IgM antibodies of 10 months follow-up (48%; 95%CI: -0.36–1.31). In long-term 12 months following up studies, the seroprevalences of IgM antibodies significantly decreased to 17% (95%CI: 0.07–0.26). The heterogeneity (I^2^) of the small study population (n < 100) group decreased to 41% after performing subgroup analysis with sample size, and it suggested that sample size probably was the source of heterogeneity.

**Fig. 8:**
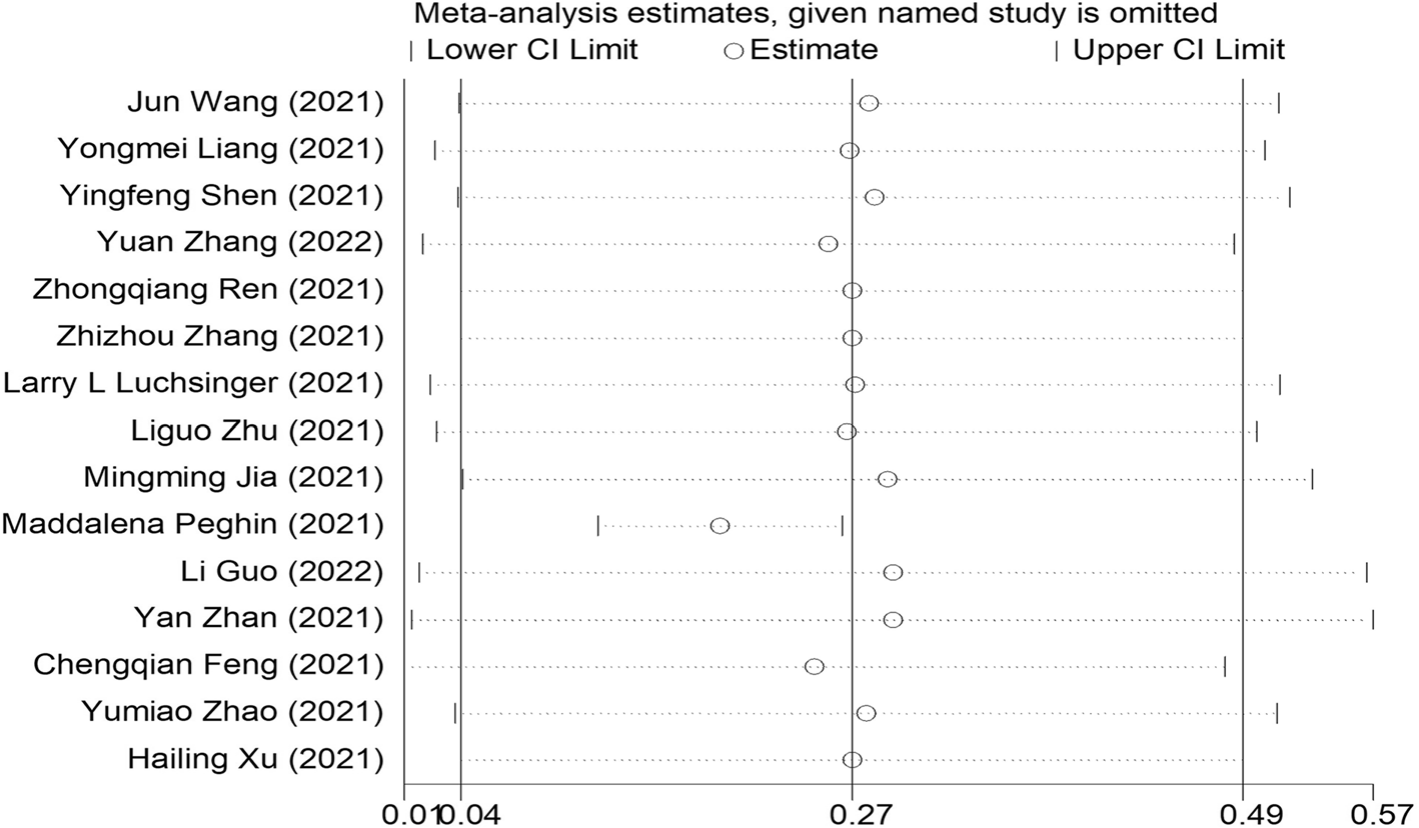
Sensitivity analysis of IgM antibodies seroprevalence. Sensitivity analysis was performed by sequential omission of individual studies

**Fig. 9:**
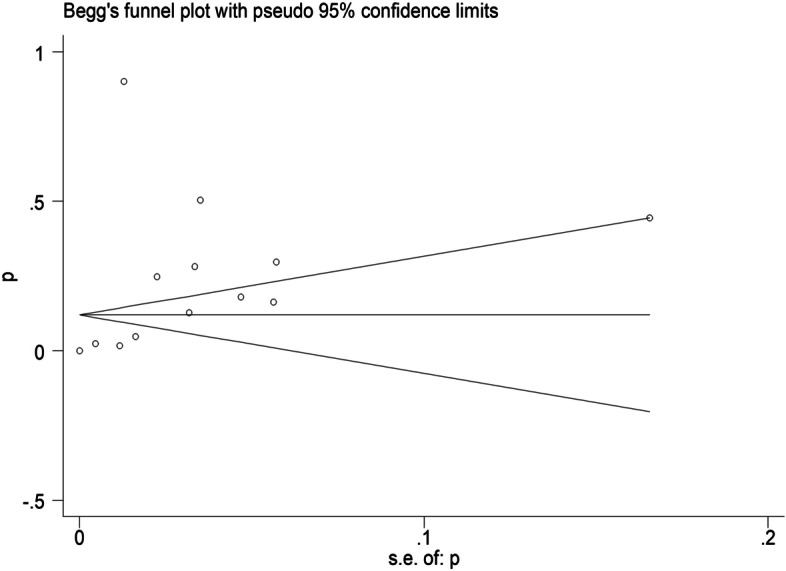
Publication bias of IgM antibodies seroprevalence

The scatter point distribution of funnel plot was slightly asymmetric (Fig. 8), but Egger’s regression test suggested no significant asymmetry of the funnel plot (*P* = 0.288), indicating no evidence of substantial publication bias in this meta-analysis (Fig. 9).

## Discussion

Establishing a robust immune response against SARS-CoV-2 coronavirus is essential for bringing the COVID-19 pandemic under control, and protecting vulnerable individuals. Despite the ample areas of research on humoral response in COVID-19, the duration of IgG and IgM antibodies in recovered COVID-19 patients is still poorly understood. In this study, the relevant literature was critically reviewed to provide an updated overview of the seroprevalences of IgG and IgM antibodies in long-term follow-up.

IgG is the most abundant antibody class in human serum. SARS-CoV-2 infection elicits a robust B cell response, resulting in the generation of detectable levels of IgG antibodies, which correlates with the development of protective immune responses and plays a crucial role in long-lasting immunity. This study revealed the seroprevalences of IgG antibodies of recovered patients with COVID-19 at long-term follow-up (follow-up time ≥ 6 months) was 66%. It may be speculated that SARS-CoV-2 exposure leads to excessive T cell activation, where terminal differentiation into effector cells predominates memory cell development, providing a long-lasting IgG titer for recovered COVID-19 patients, whether the presence of IgG antibodies protects individuals from reinfection and how long protection lasts has yet to be established [25, 33]. In addition, the quantitative relationship between viral shedding and transmissibility probability for SARS-CoV-2 IgG antibody is currently unknown [25]. Broad antibody response is observed in the early stage of SARS-CoV-2 infection, and the level of IgG antibody is exponentially increased. A previous study showed that the seroprevalence of IgG antibody was 94.29% in infected people 2 weeks after symptom onset, as the disease progresses, it can maintain a high concentration of antibody levels, and long-lasting, strong immunity response [34]. Interestingly, recent research revealed that the seroprevalence of SARS-CoV-2 IgG reached 100% approximately 240 days after symptoms onset, during the long-term follow-up, the average maintenance time was 24 months [29]. Based on the above studies, we can conclude that the titers of IgG were correlated with viral load in patients infected by SARS-CoV-2, while IgG antibody failure to clear the virus in the early stages of infection, probably led to robust stimulation of the host immune system eliciting strong and sustained immune responses, which ultimately result in disease progression.

Similar to IgG antibodies, the SARS-CoV-2 IgM antibody can be detected in the early stages of the virus infection, and it has a crucial role in virus neutralization. Typically, the level of IgM antibody lasts for a relatively short period after SARS-CoV-2 infection, which is regarded as a good and promising diagnostic biomarker for early SARS-CoV-2 infection, and it can provide a reliable reference basis for early clinical intervention. This study revealed the seroprevalences of IgM antibodies of recovered patients with COVID-19 at long-term follow-up (follow-up time ≥ 6 months) was 27%, although there was considerable heterogeneity between included studies, the results were statistically credible and reliable based on funnel plots and sensitivity analysis.

The seroprevalence of SARS-CoV-2 IgM and IgG in COVID-19 patients was 81 and 83% less than 7 days after symptom onset, both antibodies raised to 95% at 2 weeks. Approximately 8% of COVID-19 patients tested negative for IgM or IgG [35]. During the 6 to 9-month follow-up period, 25.70% of individuals were still seropositive for IgM after symptom onset, which was significantly different from other virus infections, providing a deeper understanding of IgM, whose seropositive rate gradually dropped to 55% in 9 to 10 weeks [36]. This trend is similar to our results, the seroprevalence of IgM antibody decreased to 17% in 12-month follow-up. Moreover, individuals infected by the B.1214.1 variant elicited consistently high IgG titers at 02, 03 and 06 months. Two months post vaccination with BBIP-CorV, participants showed a significant increase by × 2.5 fold of total IgG [37]. During the long-term follow-up period (162 to 282 days after symptom onset), convalescent COVID-19 patients continued to present with high IgG seropositive rates (78.13% versus 82.81%). Our results consistent with the earlier study, the seroprevalence of IgG antibody (75%) in 12 months was higher than 6 months follow-up patients (66%). Interestingly, compared to the plateaus of specific IgG against SARS 90 to 120 days after symptom onset, the decline of SARS-CoV-2 IgG was not sustained, and relatively stable phases called plateaus appeared between 162 and 282 days after symptom onset [38]. A higher antibody level may result from stronger immune response, indicating that these patients have greater activation of their immune defense during recovery, which could help clear the virus and protect patients from progression into worse conditions. These findings stress the importance of vaccination and the seroprevalence of IgM IgG should be monitored in long-term follow [29].

In our study, long-term follow-up identified the seroprevalences of IgM (66%) and IgG (27%) antibodies in recovered COVID-19 patients (follow-up time ≥ 6 months). However, due to the limited number of relevant studies, the high level of heterogeneity and the large gap in studies conducted, the findings of our study may not accurately reflect the true seroprevalence status of SARS-CoV-2 infection. Nevertheless, sequential vaccination or booster immunization is considered to be a necessary long-term strategy to sustain the fight against the pandemic. The main limitations of the current study are: (I) The levels of IgM and IgG antibodies in the different conditions of COVID-19 (asymptomatic infection, mild, common, severe, and critically severe) need to be evaluated in long-term follow-up study in order to be able to claim effects. (II) This study did not assess the seroprevalences of anti-SARS-CoV-2 IgA, RBD Ab, and Nab antibodies, which play an important role in the local mucosal immunity. (III) The cut-off values and measuring techniques varies between included studies, which might affect the reliability of systematic review conclusions. (IV) Because of high heterogeneity and limited studies in this meta-analysis, future high-quality researches are still needed to confirm these outcomes.

## Conclusions

In conclusion, the results of our meta-analysis showed that the seroprevalences of IgM antibody decreased and IgG antibody was higher than 6 months follow-up patients when compared to 12 months following up studies. In the future, large-scale, long-term research should be devoted to confirming the results of our meta-analysis. Further assessing the duration, effect and ability to resist reinfection of anti-SARS-CoV-2 IgA, RBD Ab, and Nab antibodies in COVID-19 recovered patients is needed, and clarify the underlying mechanisms of their relationship. Sequential vaccination or booster immunization is considered to be a necessary long-term strategy to sustain the fight against the pandemic.

## Data Availability

All data analyzed in this study are included in this article.

## References

[1] Liu Y, Ye Q (2022). Safety and efficacy of the common vaccines against COVID-19. Vaccines.

[2] Chan DK (2022). Rapid deployment of multiple tactics to address severe acute respiratory syndrome Coronavirus 2 vaccine uptake in healthcare employees with a focus on those who identify as black, indigenous, and people of color. Open Forum Infect Dis.

[3] Munster VJ, Koopmans M, van Doremalen N, van Riel D, de Wit E (2020). A novel coronavirus emerging in China - key questions for impact assessment. N Engl J Med.

[4] Pušnik J (2022). Persistent maintenance of intermediate memory B cells following SARS-CoV-2 infection and vaccination recall response. J Virol.

[5] Prasithsirikul W (2022). ChAdOx1 nCoV-19 immunogenicity and immunological response following COVID-19 infection in patients receiving maintenance hemodialysis. Vaccines.

[6] Wang X (2020). Accurate diagnosis of COVID-19 by a novel immunogenic secreted SARS-CoV-2 orf8 protein. mBio.

[7] Peghin M (2021). The fall in antibody response to SARS-CoV-2: a longitudinal study of a symptomatic to critically Ill patients up to 10 months after recovery. J Clin Microbiol.

[8] Moher D, Liberati A, Tetzlaff J, Altman DG (2009). Preferred reporting items for systematic reviews and meta-analyses: the PRISMA statement. BMJ (Clinical research ed.).

[9] Slim K (2003). Methodological index for non-randomized studies (minors): development and validation of a new instrument. ANZ J Surg.

[10] Mirel B (2013). Studying PubMed usages in the field for complex problem solving: implications for tool design. J Am Soc Inform Sci Technol.

[11] Luo D, Wan X, Liu J, Tong T (2018). Optimally estimating the sample mean from the sample size, median, mid-range, and/or mid-quartile range. Stat Methods Med Res.

[12] Wan X, Wang W, Liu J, Tong T (2014). Estimating the sample mean and standard deviation from the sample size, median, range and/or interquartile range. BMC Med Res Methodol.

[13] Wang J, H. L. Analysis of the results of dynamic serum antibody testing in 43 patients with COVID-19. J Pathog Biol. 2021;16:1143–1147+1152. 10.13350/j.cjpb.211006.

[14] Zhao B, Zhong J, Zhou M (2021). Analysis of the SARS-CoV-2 specific antibodies and immune function of COVID-19 patients one year after discharge. Chin J Clin Thorac Cardiovasc Surg.

[15] Liang Y, Yue Y, Li L (2021). Serum antibody levels in COVID-19 patients six months after hospital discharge. Shanghai J Prevent Med.

[16] Shen Y, Ba Y, Hu Y (2021). Analysis of relationship between dynamic changes of serum SARS-CoV-2 specific IgM/IgG and patient immunity in recovery stage. Acta Med Univ Sci Technol Huazhong.

[17] Zhang Y, Ding J & J, L. Detection and analysis of neutralizing antibodies against SARS-CoV-2 in convalescent plasma donors. Chin J Blood Transfusion. 2022;35:245–249, 10.13303/j.cjbt.issn.1004-549x.2022.03.003.

[18] Xu H, Z. J., Luo Y. The observation of serum specific IgM/IgG levels in COVID-19 cured patients after 1 year. Prim Med Forum. 2021;25:3589–3591. 10.19435/j.1672-1721.2021.25.016.

[19] Jia M, Liu L, Cai X (2021). Changes of antibodies in COVID-19 patients: a 10-month prospective study. Med J Wuhan Univ.

[20] Zhong R, Zhang T & Y, L. Study on the progression and prognosis of COVID-19 patients in Zhongshan city. Chin Commun Doctors. 2021;37:46–47+50.

[21] Zhang Z & H, L. Analysis of the detection results of serum antibody levels in patients with COVID-19 after recovery. J Med Theor Prac. 2021;34:2496–2498, 10.19381/j.issn.1001-7585.2021.14.061.

[22] Zhan Y (2021). SARS-CoV-2 immunity and functional recovery of COVID-19 patients 1-year after infection. Signal Transduct Target Ther.

[23] Achiron A (2021). SARS-CoV-2 antibody dynamics and B-cell memory response over time in COVID-19 convalescent subjects. Clin Microbiol Infect.

[24] Sakhi H (2021). Kinetics of anti-SARS-CoV-2 IgG antibodies in hemodialysis patients six months after infection. J Am Soc Nephrol.

[25] Yao L (2021). Persistence of antibody and cellular immune responses in Coronavirus disease 2019 patients over nine months after infection. J Infect Dis.

[26] Masiá M (2021). SARS-CoV-2 seroconversion and viral clearance in patients hospitalized with COVID-19: viral load predicts antibody response. Open Forum Infect Dis.

[27] Feng C (2021). Protective humoral and cellular immune responses to SARS-CoV-2 persist up to 1 year after recovery. Nat Commun.

[28] Zhao Y (2021). Follow-up study on COVID-19 survivors one year after discharge from hospital. Int J Infect Dis.

[29] Zhu L (2021). Kinetics of SARS-CoV-2 specific and neutralizing antibodies over seven months after symptom onset in COVID-19 patients. Microbiol Spectr.

[30] Guo L (2022). SARS-CoV-2-specific antibody and T-cell responses 1 year after infection in people recovered from COVID-19: a longitudinal cohort study. Lancet Microbe.

[31] Schiffner J (2021). Long-term course of humoral and cellular immune responses in outpatients after SARS-CoV-2 infection. Front Public Health.

[32] Luchsinger LL (2020). Serological assays estimate highly variable SARS-CoV-2 neutralizing antibody activity in recovered COVID-19 patients. J Clin Microbiol.

[33] Moser D (2021). COVID-19 impairs immune response to Candida albicans. Front Immunol.

[34] Poland GA, Ovsyannikova IG, Kennedy RB (2020). SARS-CoV-2 immunity: review and applications to phase 3 vaccine candidates. Lancet (London, England).

[35] Qin X (2021). The seroprevalence and kinetics of IgM and IgG in the progression of C OVID-19. BMC Immunol.

[36] Fu Y (2021). Dynamics and correlation among viral positivity, seroconversion, and disease severity in COVID-19: a retrospective study. Ann Intern Med.

[37] Batchi-Bouyou AL (2022). Assessment of neutralizing antibody responses after natural SARS-CoV-2 infection and vaccination in congolese individuals. BMC Infect Dis.

[38] Wu L-P (2007). Duration of antibody responses after severe acute respiratory syndrome. Emerg Infect Dis.

